# The Unique Role of the ECERIFERUM2-LIKE Clade of the BAHD Acyltransferase Superfamily in Cuticular Wax Metabolism

**DOI:** 10.3390/plants6020023

**Published:** 2017-06-13

**Authors:** Tegan M. Haslam, Wesley K. Gerelle, Sean W. Graham, Ljerka Kunst

**Affiliations:** Department of Botany, University of British Columbia, Vancouver, BC V6T 1Z4, Canada; tegan.haslam@botany.ubc.ca (T.M.H.); wesley.gerelle@gmail.com (W.K.G.); swgraham@mail.ubc.ca (S.W.G.)

**Keywords:** cuticle, very-long-chain fatty acids, *eceriferum*, BAHD acyltransferase, elongation, evolution

## Abstract

The elongation of very-long-chain fatty acids is a conserved process used for the production of many metabolites, including plant cuticular waxes. The elongation of precursors of the most abundant cuticular wax components of some plants, however, is unique in requiring ECERIFERUM2-LIKE (CER2-LIKE) proteins. CER2-LIKEs are a clade within the BAHD superfamily of acyltransferases. They are known to be required for cuticular wax production in both Arabidopsis and maize based on mutant studies. Heterologous expression of Arabidopsis and rice CER2-LIKEs in *Saccharomyces cerevisiae* has demonstrated that they modify the chain-length specificity of elongation when paired with particular condensing enzymes. Despite sequence homology, CER2-LIKEs are distinct from the BAHD superfamily in that they do not appear to use acyl transfer activity to fulfill their biological function. Here, we review the discovery and characterization of CER2-LIKEs, propose several models to explain their function, and explore the importance of CER2-LIKE proteins for the evolution of plant cuticles.

## 1. Very-Long-Chain Fatty Acids and Cuticular Wax Metabolism

Lipid barriers are essential across all domains of life. For example, lipid bilayers serve as barriers that enable the organization and regulation of cellular processes. On a larger scale, lipids form barriers that protect and seal specific tissues and organs. One such barrier is the cuticle, which coats the primary aerial surfaces of land plants. The cuticle consists of two lipidic components: cutin and cuticular wax. Cutin is a polymer of oxidized long-chain fatty acid derivatives and glycerol linked by ester bonds (reviewed in [1]). Cutin forms a matrix across the plant surface that provides resistance to pathogens and herbivores, and prevents the fusion of epidermal cells. The cutin polymer is embedded and overlaid with cuticular wax, which is a mixture of very-long-chain fatty acids (VLCFAs) and their derivatives. Cuticular wax restricts transpiration, allowing plants to retain water and thrive in terrestrial environments. Wax structure and texture can also affect the reflection of light off of the plant surface, and facilitate or impede insect movement on the plant.

**Figure 1:**
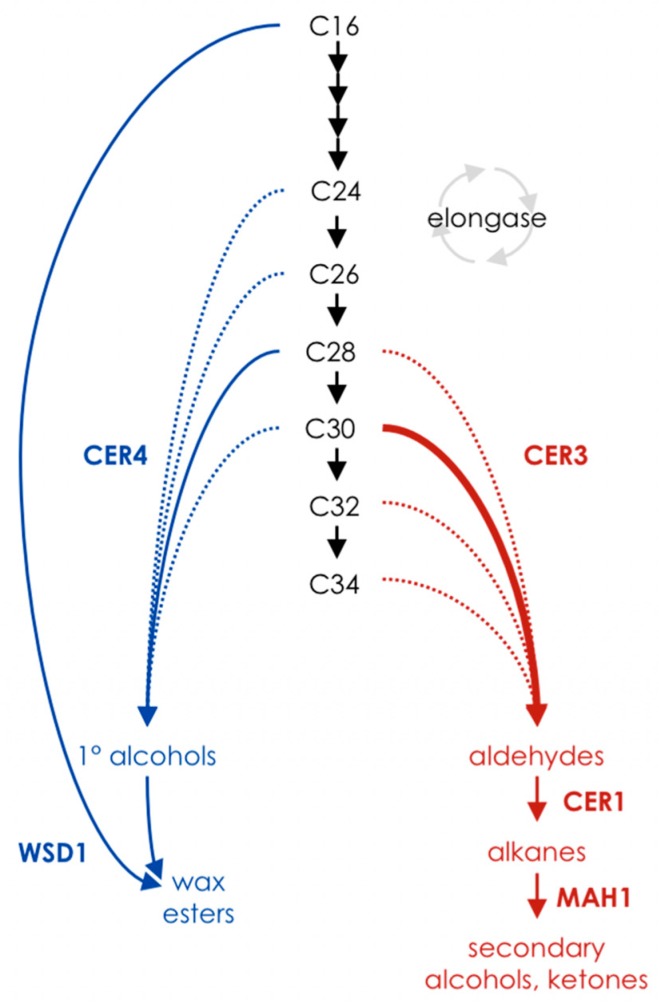
Schematic of cuticular wax biosynthesis in Arabidopsis. Very-long-chain fatty acids (VLCFAs) are lengthened in two-carbon increments (black), and derivatized by one of two pathways. The acyl-reduction pathway produces primary alcohols, a portion of which are esterified to fatty acids to make wax esters (blue). The alkane-forming pathway produces aldehyde intermediates, from which a carbonyl group is lost to form an alkane; alkanes may subsequently undergo mid-chain oxidation to produce secondary alcohols and ketones (red). CER: ECERIFERUM; WSD: bifunctional wax ester synthase/diacylglycerol acyltransferase; MAH1: mid-chain alkane hydroxylase.

Wax synthesis occurs in the epidermis and begins with the elongation of long-chain fatty acids to very-long-chain fatty acids. A fraction of these VLCFAs are secreted to the apoplast directly, and the rest are processed by either an acyl-reduction or alkane-forming pathway prior to their deposition in the cuticle (reviewed in [2]). The acyl-reduction pathway converts very-long-chain acyl-CoAs to primary alcohols, which can subsequently be esterified to fatty acids to form wax esters. The alkane-forming pathway generates aldehydes and alkanes from acyl-CoAs by a redox-dependent mechanism. A portion of the alkanes synthesized in this pathway is modified by mid-chain oxidation to make secondary alcohols and ketones (Figure 1).

Genetic studies in Arabidopsis have predominated in the field of cuticular wax metabolism over the last two decades, benefiting from the genetic toolkit available for this model organism. More recently, there has also been rapid progress in the identification and characterization of genes required for the synthesis of cuticular wax in barley, rice, tomato, and many other plants. This trend will doubtless continue as more genomic sequences become available, and with the ongoing, rapid development of gene editing tools. Because cuticular wax composition varies among plants, work beyond Arabidopsis has been important in uncovering clade-specific intricacies of wax biosynthesis, as well as identifying diverse triterpenoid and phenolic components that also accumulate in the cuticle. One feature of cuticular wax composition that is consistent across many flowering plant clades is the chain length of wax components; most are derived from fatty acids 26–34 carbons in length. Other metabolic pathways that require VLCFAs, such as sphingolipid, suberin, or oil synthesis, use shorter acyl chain lengths. Elongation is therefore important for regulating the production of cuticular waxes. This review will describe how the extension of cuticular wax precursors is also biochemically distinct from elongation to shorter chain lengths. Aside from the core elongation machinery involved in the synthesis of all VLCFAs, an additional protein family, CER2-LIKEs, uniquely contributes to the formation of VLCFA cuticular wax precursors. In the following section, we will briefly describe the core components of the elongase complex, and summarize several interesting observations and incongruities regarding the elongation process. With this introduction, we aim to set the context for the discovery and characterization of CER2-LIKEs.

## 2. The Fatty Acid Elongase

**Figure 2:**
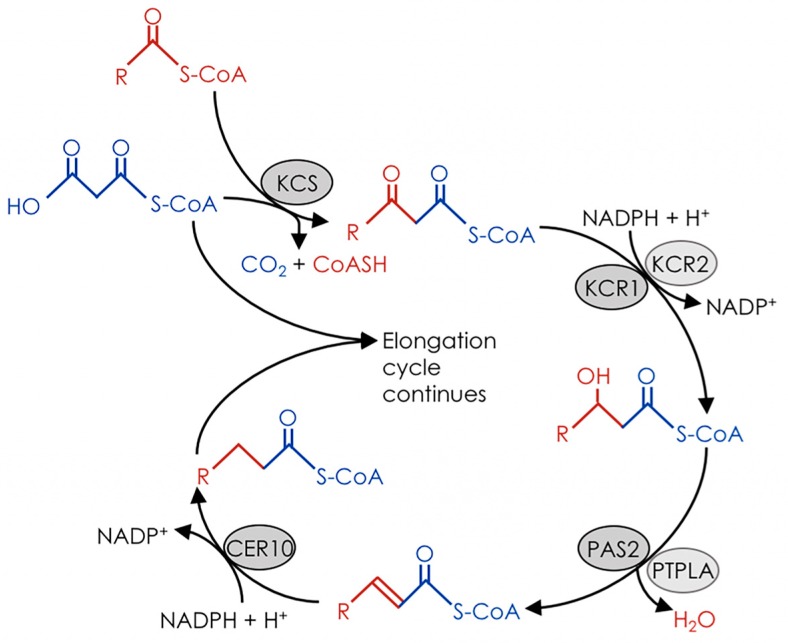
Schematic of fatty acid elongation. Malonyl-CoA (blue) and an acyl-CoA primer (red) are condensed by a ketoacyl-CoA synthase (KCS) to produce a β-ketoacyl-CoA. The β-ketoacyl-CoA is reduced by a β-ketoacyl-CoA reductase (KCR) to yield a β-hydroxyacyl-CoA, which in turn is dehydrated by a β-hydroxyacyl-CoA dehydratase (HCD/PAS2) to produce an enoyl-CoA. The enoyl-CoA is reduced by an enoyl-CoA reductase (ECR/CER10) to give an acyl-CoA that is two carbons longer than the initial acyl-CoA used as a primer. The acyl-CoA product can then be used as a primer for the same reaction sequence, allowing for repeated elongation of VLCFAs in two-carbon units.

The fatty acid elongase is a membrane-bound protein complex localized to the endoplasmic reticulum (ER). Analogous to the soluble fatty acid synthase of the plastid stroma, the elongase extends growing fatty acid chains by iterative addition of two-carbon units. At least four enzymatic activities are required for each elongation cycle. The first reaction is catalyzed by a condensing enzyme, or keto-acyl-CoA synthase (KCS), which condenses the acyl-CoA (*n*) substrate with malonyl-CoA. The β-keto group of the β-keto-acyl-CoA (*n* + 2) product is then reduced to an alcohol by a β-ketoacyl-CoA reductase (KCR), which is dehydrated to an enoyl by a hydroxyacyl-CoA dehydratase (HCD), and finally reduced by an enoyl-CoA reductase (ECR) to generate the straight-chain *n* + 2 acyl-CoA product (Figure 2; reviewed in [3]). While condensing enzymes have specificity for particular chain lengths [4], the KCR, HCD, and ECR enzymes appear to function with substrates of all chain lengths taken up by the complex. Because of this requirement for different condensing enzymes for specific fatty acid chain lengths, KCSs are typically encoded by large gene families with unique expression patterns, whereas the “generalist” components of the elongase are encoded by one or two genes that are broadly expressed. While there are multiple lines of evidence that support this model, there have also been indications that it is an oversimplification of more elaborate biosynthetic machinery that exists in nature.

Recent work has cast some doubt on the notion that the generalist functions of the elongase are carried out by singular proteins. Arabidopsis *PAS2* has been annotated as the hydroxyacyl-CoA dehydratase based on mutant studies and on heterologous expression in yeast [5]. A second Arabidopsis gene, *PROTEIN TYROSINE PHOSPHATASE-LIKE* (*PTPLA*), has since been shown to complement the slow growth and VLCFA-deficient phenotypes of the yeast hydroxyacyl-CoA dehydratase mutant *Tet-PHS1* [6]. However, *PTPLA* expressed in Arabidopsis using the *PAS2* promoter cannot complement the *pas2* mutant phenotype. Characterization of the *ptpla* mutant revealed that although it has none of the obvious growth defects that characterize the *pas2* mutant, it accumulates hydroxyacyl-CoAs, which is a key feature of HCD deficiency. Oddly, the mutant also accumulates more VLCFAs than wild-type plants. *PTPLA* expression patterns are distinct from those of the core elongase components *PAS2*, *KCR1,* and *CER10* [6]. *PTPLA* is co-expressed with an unusual β-ketoacyl-CoA reductase, *KCR2*. Analogous to *PTPLA*, *KCR2* was previously investigated due to its sequence similarity to *KCR1*, but the *kcr2* mutant has no obvious phenotype, and expression of *KCR2* driven by the *KCR1* promoter cannot complement *KCR1*-downregulated plants [7]. Based on these observations, it was proposed that two separate elongase systems exist in plants, expressed in different cell types; one with PAS2 and KCR1, and one with PTPLA and KCR2 [6]. What physiological purpose this elaboration of fatty acid elongation would serve is unclear, and a mechanistic explanation for the conflicting phenotypes of the *ptpla* mutant remains elusive.

Another glaring gap with regard to our understanding of elongation is that the stoichiometry of the elongase components within the complex is completely unknown. The simplest model is a heterotetramer, with one condensing enzyme and one of each of the generalist enzymes in a single complex. Different elongase heterotetramers could have different condensing enzymes, with substrate passed from one entire complex to another to generate longer and longer acyl-CoA chain lengths. Alternatively, multiple condensing enzymes could function with the same set of generalist components, allowing for elongation of a given fatty acid from start to finish. A suite of condensing enzymes could be swapped in and out of the complex as longer and longer fatty acids are generated, or multiple condensing enzymes could simultaneously associate with the same complex. Alternatively, elongase complexes could exist as larger aggregations with multiple copies of any or all of their protein components, including both condensing enzymes and the generalist enzymes.

Perhaps the most intriguing complexity of elongation in plants is that two non-homologous families of condensing enzymes have been identified. Fatty Acid Elongation 1 (FAE1)-like KCSs are homologous to the first Arabidopsis condensing enzyme discovered, FATTY ACID ELONGATION 1, which is involved in seed oil biosynthesis. FAE1-likes have been extensively characterized in Arabidopsis, and are known to have diverse and important roles in plant metabolism (reviewed in [8]). On the other hand, plant Elongation Defective (ELO)-like condensing enzymes are homologous to yeast ELOs and animal ELOVLs (Elongation of Very-Long-Chain Fatty Acids). To date, no function has been ascribed to the four *ELO*-likes identified in the Arabidopsis genome, and the function of these genes in flowering plants remains a mystery. However, some *ELO*-like genes have been characterized in bryophytes. Three cDNAs encoding *ELO* homologs have been isolated from the moss *Physcomitrella patens*, and mutant analysis and heterologous expression in yeast have demonstrated that they elongate polyunsaturated VLCFAs [9,10]. Polyunsaturated VLCFAs are incorporated into diverse membrane lipids in bryophytes.

The function of KCSs as rate-limiting, substrate-specific components of the elongase is supported by mutant characterizations (summarized in [8]), heterologous expression of *FAE1* in tobacco and in yeast [4], and expression of many other plant KCSs in yeast [11,12,13,14]. Strikingly, these heterologous expression experiments have revealed that none of the 21 identified KCSs of Arabidopsis can efficiently generate VLCFAs beyond 28 carbons. As VLCFAs in excess of 28 carbons make up the bulk of cuticular waxes on Arabidopsis, and indeed on many plants surveyed to date, this is a conspicuous deficiency. Identification and characterization of Arabidopsis *ECERIFERUM2-LIKEs* (*CER2-LIKE*s) has revealed that proteins encoded by these genes play a key role in modifying the activity of specific condensing enzymes, and enable the production of VLCFA precursors of cuticular waxes longer than 28 carbons in length.

## 3. Identification and Characterization of *ECERIFERUM2-LIKE* (*CER2-LIKE*) Genes

*Eceriferum2* (*cer2*) was among the most severe wax-deficient mutants identified in a visual screen for glossy stem wax phenotypes in ethylmethane sulfonate (EMS)-induced and irradiated mutant populations of Arabidopsis [15]. Several more *cer2* mutant alleles were isolated in a subsequent screen for wax deficiency [16]. Characterization of the *cer2-4* allele [16] revealed that it specifically lacks stem cuticular wax components longer than 28 carbons and accumulates more 26-carbon wax components than wild type. This phenotype strongly suggests that *CER2* could have a role in the elongation of fatty acids beyond 28 carbons. As cuticular waxes derived from fatty acids 30 carbons and longer make up over 80% of the total wax of wild-type Arabidopsis stems, it is not surprising that the *cer2* mutant stems appear glossy and green compared to the glaucous wild type [16]. Map-based cloning of the *CER2* gene revealed that it is homologous to BAHD acyltransferases [17,18]. Given the current model of fatty acid elongation, however, it was not obvious why an acyltransferase would be required for elongation of particular chain lengths of acyl-CoAs.

Because heterologous expression in yeast has been a useful tool for characterizing the core components of the plant fatty acid elongase, expression of CER2 in yeast was an obvious approach to decipher the role of this protein. CER2 expressed in yeast had little effect on fatty acid metabolism, however, even when provided with its presumed 28-carbon fatty acid substrate by co-expression with the condensing enzyme LfKCS45, which produces ample 28-carbon VLCFAs in yeast cells. CER6, a condensing enzyme known to have a central role in cuticular wax production in Arabidopsis [19], was previously reported to elongate VLCFAs only to 28 carbons when expressed in yeast cells. Remarkably, when CER2 was co-expressed with CER6, it modified elongation such that 30-carbon product accumulated. This result demonstrates that CER2 is indeed a component of the fatty acid elongation machinery, and that CER6 and CER2 are sufficient for the production of 30-carbon precursors of stem cuticular wax. Additionally, this experiment showed that *CER*2 specifically requires CER6 to carry out its function, as CER2 could not function alongside LfKCS45 [20].

A gene with high sequence identity to Arabidopsis *CER2*, named *GLOSSY2* (*GL2*), was identified in *Zea mays* (maize) [21,22]. Cuticular wax composition varies between leaf developmental stages in maize, with juvenile leaves having waxes mainly 32 carbons in length, and adult leaves having waxes mostly 30 carbons in length. The cuticular wax of juvenile leaves of the *gl2* mutant is made up predominantly of 30-carbon wax monomers, indicating that *GL2* is required for wax precursor elongation from 30 to 32 carbons, an analogous role to Arabidopsis *CER2* in the elongation of wax precursors from 28 to 30 carbons.

Four additional genes with high sequence identity to *CER2* have also been identified and characterized in Arabidopsis [20,23,24,25]. Characterization of these *CER2-LIKE*s has provided insight into the function of this gene family. Different *cer2-like* single, double, and triple mutants have modified wax profiles on different organs [20,23,24]. Some of the *cer2-like* mutants are also male-sterile under low humidity [24,26]. Loss of fertility in dry conditions is a characteristic phenotype associated with pollen coat modifications; given that pollen coat contains very similar acyl lipids to cuticular wax, this additional phenotype of *cer2-like* mutants is not surprising.

When each of the CER2-LIKEs is co-expressed in yeast with the CER6 condensing enzyme, they have unique effects on fatty acid elongation. For example, while CER6 and CER2 elongate fatty acids to 30 carbons, CER6 and CER2-LIKE1 elongate fatty acids to 34 carbons. CER2, CER2-LIKE1, and CER2-LIKE2 have also been co-expressed with other condensing enzymes, but were only reported to function alongside CER6 and its close homolog, CER60 [24]. Recent characterization of the Arabidopsis condensing enzyme KCS16, however, demonstrated that co-expression of KCS16 with CER6 and CER2-LIKE1 in yeast cells resulted in the accumulation of 36- and 38-carbon VLCFAs. KCS16 paired with just CER6 only accumulated VLCFAs up to 28 carbons in yeast, similar to CER6 alone, and KCS16 paired with just CER2-LIKE1 had the same VLCFA profile as untransformed yeast cells [27]. When CER6, CER2-LIKE1, and KCS16 are all co-expressed, it is unclear whether 34-carbon substrate is produced by the CER6 and CER2-LIKE1 pair and subsequently elongated by KCS16, or whether KCS16 and CER2-LIKE1 carry out the final elongation steps together. What conditions must be met for a condensing enzyme to function with a CER2-LIKE is unknown, and in the absence of this knowledge, it is impossible to predict which other condensing enzymes might have the capacity to function with CER2-LIKEs. Notably, a CER2 homolog was recently identified in rice and was demonstrated to function alongside the rice condensing enzyme WAX CRYSTAL-SPARSE LEAF 4 (WSL4) when co-expressed in yeast cells [28]. While rice *cer2* mutants were not investigated in this study, extensive biochemical experiments were carried out, which are discussed in the following section of this review.

Ectopic expression of Arabidopsis *CER2-LIKE1* has shed light on the physiological importance of the seemingly subtle changes in wax chain length caused by *CER2-LIKE*s. *CER2-LIKE1* is normally expressed in leaves and siliques, and its mutant phenotype and activity in yeast indicate that it elongates VLCFAs to 34 carbons in length. When *CER2-LIKE1* is ectopically expressed in stems using either the *35S* promoter [23] or the epidermis-specific *CER6* promoter [24], *CER2-LIKE1* modifies the chain-length profile of stem waxes such that it resembles that of leaf waxes. That is, it has proportionally less wax derived from 30-carbon fatty acids and accumulates more wax derived from 32- and 34-carbon fatty acids. Surprisingly, although the overall wax load of stems expressing *CER2-LIKE1* was not substantially different from wild type, and the relative amounts of different types of waxes did not vary considerably, the stems appeared glossy and green, similar to wax-deficient mutants. This suggests that cuticular wax chain length has an effect on the formation of epicuticular wax structures. The texture of plant cuticles determines the degree of water repellency and particle adhesion, light reflection, and may facilitate or impede the movement of insects on the plant surface; the formation of epicuticular wax crystals is, therefore, physiologically important. In light of these experiments, it is of interest to assess how wax chain length varies in different plant species, as well as how widely distributed *CER2-LIKE* genes are throughout the plant kingdom.

One *CER2-LIKE* gene from Arabidopsis, *CER2-LIKE4*, was recently shown by two research groups to have an atypical function compared to the other homologs [29,25]. Characterization of *CER2-LIKE4* overexpression lines revealed that although they had a glossy, green appearance similar to wax-deficient mutants, their total stem wax load did not differ from the wild type. Stem wax compositional analyses revealed that the overexpressors had increased amounts of waxes derived from 28-carbon fatty acids, namely 28-carbon primary alcohol and aldehyde, and decreased amounts of longer wax components, most noticeably the 29-carbon alkane, secondary alcohol, and ketone that predominate in the wax of wild-type stems [29]. While no mutant has been characterized, co-expression of CER6 and CER2-LIKE4 in yeast cells has shown that CER2-LIKE4 does not extend the chain-length specificity of CER6, as all the other Arabidopsis CER2-LIKE proteins do. Relative to the fatty acid profile of yeast expressing CER6 alone, cells co-expressing CER6 and CER2-LIKE4 accumulated less 26-carbon fatty acid, whereas the amount of 28-carbon fatty acid was unchanged [25]. Together, these results suggest that CER2-LIKE4 could be a regulator of CER6 activity, but its role is unclear. A null *cer2-like4* mutant will certainly be required for further studies of this gene.

## 4. Biochemical Function of *CER2*

The paired activity of CER2 and CER6 observed in yeast cells suggested that these proteins might physically interact. Recent work on Arabidopsis CER2 and on its rice homolog OsCER*2* have provided evidence that they both, indeed, interact with their condensing enzyme partners. OsCER*2* was identified by co-immunoprecipitation with the condensing enzyme WSL4, and interaction between the two proteins was confirmed in a yeast-two-hybrid assay [28]. Interaction between Arabidopsis CER2 and the condensing enzyme CER6 was demonstrated by co-immunoprecipitation and by a split-luciferase assay in *Nicotiana benthamiana*, which also revealed that CER2 is in close physical proximity to the other core protein components of the fatty acid elongase [25].

Despite clear demonstration that CER2-LIKEs affect VLCFA elongation, the exact biochemical function of this protein family in the elongation process remains elusive. Based on sequence homology, CER2 has been annotated as a BAHD acyltransferase. BAHD enzymes transfer an acyl group from a CoA-thioester to either an alcohol or amine acyl acceptor, generating either an ester or amide bond, respectively. Two conserved motifs have been described for BAHDs: carboxy-terminal DFGWG, which is predicted to have a function in retaining structural stability of the enzyme but is not present in all BAHDs, and HXXXD, which catalyzes the acyl transfer reaction [30]. Arabidopsis CER2-LIKEs all lack the DFGWG motif. The fact that CER2 and CER2-LIKE1 localize to the ER membrane [20,24], while other characterized BAHDs are soluble enzymes [30,31,32], suggests that the stabilizing DFGWG motif may not be required when these proteins associate with the ER.

**Figure 3:**
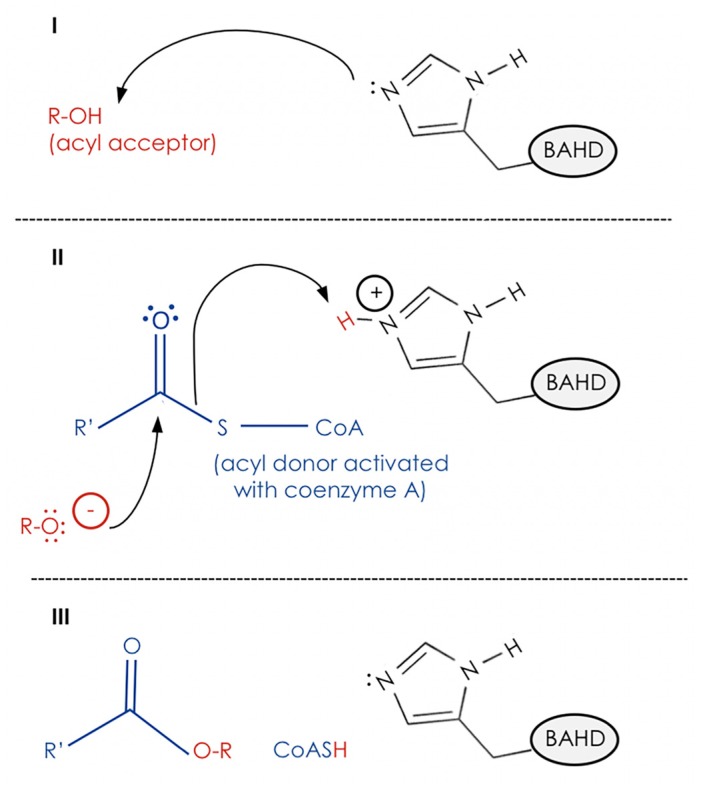
Simplified schematic of BAHD acyl transfer activity. First (I), the acyl acceptor is deprotonated by a catalytic histidine residue on the BAHD. Second (II), the electronegative acyl acceptor carries out a nucleophilic attack on the carbonyl carbon of the acyl-CoA, the acyl donor. This is proposed to form a tetrahedral intermediate (not shown). Finally (III), the new ester bond between the acyl donor and acceptor is created with the release of CoA.

The role of the HXXXD motif has been demonstrated with many BAHD acyltransferases; site-directed mutagenesis experiments on anthocyanin malonyltransferase of *Salvia splendens* [33], vinorine synthase of *Rauvolfia serpentine* [34], and hydroxycinnamoyltransferases of *Coffea canefora* [35] and *Sorghum bicolor* [36] have shown that the histidine residue within this motif is essential for catalytic activity. The histidine deprotonates the acyl acceptor substrate, creating a nucleophile that attacks the carbonyl carbon of the acyl-CoA substrate, resulting in the release of CoASH and formation the ester or amide product (Figure 3). The role of the conserved histidine is supported by the crystal structures of vinorine synthase from *Rauvolfia* and anthocyanin malonyltransferase from *Chrysanthemum*, in which this amino acid is positioned at the junction of the acyl donor and acyl acceptor binding sites [37,38]. However, both CER2-LIKE1 and CER2-LIKE4 lack a histidine in their predicted HXXXD motifs. Genetic analysis of a null *cer2-like1* mutant [23,24] indicated that despite this, CER2-LIKE1 has an analogous role to CER2 in VLCFA elongation. Site-directed mutagenesis of H_166,_ the predicted catalytic histidine residue of CER2, revealed that it is not required for CER2 to contribute to the elongation of wax precursors, as H_166_A and H_166_N mutant alleles could fully complement the *cer2* wax-deficient phenotype [24]. H_172_A and H_172_N mutations of OsCER*2*, which mirror the H_166_A and H_166_N alleles of Arabidopsis CER2, also did not affect the elongation activity of OsCER*2* or its interaction with the condensing enzyme WSL4 [28]. Taken together, results of these two independent studies suggest that the catalytic mechanism established for other BAHD acyltransferases cannot be extrapolated to describe CER2-LIKE function. Interestingly, some substitutions within the HXXXD motif of OsCER*2*, H_172_D, D_176_A, and D_176_H, did impair both elongation activity and interaction with WSL4 [28]. *In silico* structural modelling of an alcohol acyltransferase from mountain papaya (*Vasconcellea pubescens*) (VpAAT) has provided evidence that, in addition to catalytic activity, the HXXXD motif of this BAHD influences protein structure. Homology-based models of wild-type and modified sequences showed that a solvent channel present in the wild-type VpAAT model was collapsed when the aspartic acid of the HXXXD motif was substituted with alanine, glutamic acid, or asparagine. In contrast, substitution of the predicted catalytic histidine residue did not change the architecture of the solvent channel. The authors concluded that the aspartic acid residue of the conserved HXXXD motif is not strictly catalytic, but plays a structural role that is essential for supporting the channel, and positions the substrates and histidine residue for catalysis [39]. Collectively, these experiments suggest that some substitutions within the HXXXD motif of CER2-LIKEs can affect protein structure and thereby influence function. However, the catalytic activity characteristic of this motif (i.e., deprotonation of the acyl acceptor by a histidine residue acting as a general base) is not relevant to the role of CER2-LIKEs in wax metabolism. This, and the absence of an obvious purpose of an acyl transfer reaction for the elongation of VLCFAs, invites skepticism as to whether CER2-LIKEs have acyl transfer activity at all.

**Figure 4:**
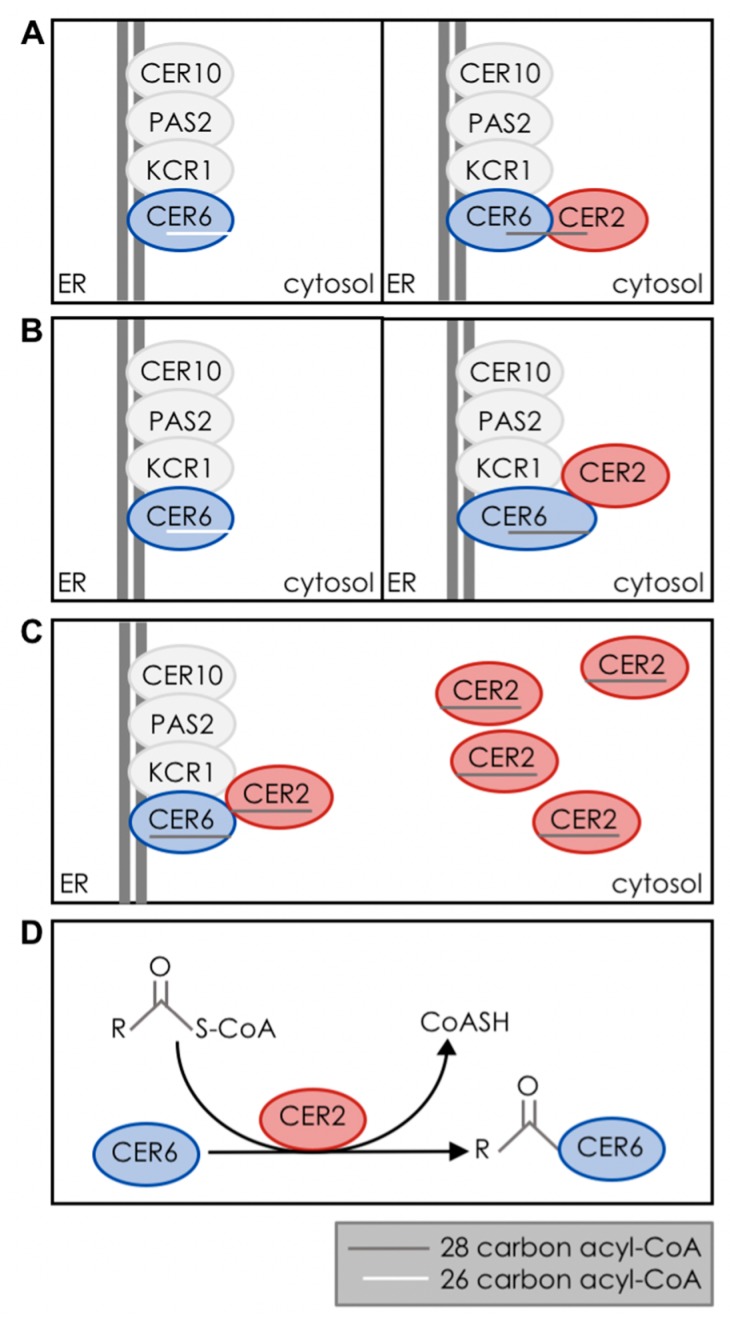
Models of paired CER6-CER2 activity. (**A**) Active site extension. A hypothetical acyl-CoA binding site in CER2 could physically extend the substrate-binding pocket of CER6; (**B**) Allosteric modulation. Protein-protein interaction between CER2 and CER6 could modify the structure of the substrate-binding pocket of CER6, enabling it to accept longer acyl chains; (**C**) Substrate binding and concentration. CER2 could provide acyl-CoAs to the fatty acid elongase; (**D**) Thioesterase activity. CER2 could act as a thioesterase, providing free fatty acid to load onto the active-site cysteine of CER6.

Although the biochemical function of CER2-LIKEs remains unknown, several models can be proposed to guide future investigation of this protein family (Figure 4). One possibility is that the acyl-CoA binding capacity of KCSs restricts the maximum length of elongation, and that interaction with CER2-LIKEs enables KCSs to use longer substrates. To date, the acyl-CoA substrate specificity of plant KCS enzymes has primarily been investigated using molecular genetic approaches. Domains affecting specificity have been identified in sequence swaps between orthologous genes [40], and residues that affect substrate specificity have been found by quantitative trait locus (QTL) mapping in wild accessions [41]. The structure of the acyl-CoA binding pocket of KCSs has only been described using homology-based models [8]. Therefore, while the sizes of substrate binding pockets of different KCSs can be compared based on models and predictions, their absolute dimensions are unknown. It is not clear whether the substrate-binding pocket of CER6 can support binding of 26-, 28-, 30-, or 32-carbon acyl-CoAs. If substrate binding by condensing enzymes is limiting, CER2-LIKEs could modify the substrate specificity of condensing enzymes via allosteric interaction. CER2-LIKE binding could alter the tertiary structure of the condensing enzyme in such a way as to reshape or expand the size of the substrate-binding pocket. Different CER2-LIKEs could interact differently with their condensing enzyme partner to account for the unique product specificities of CER2-LIKEs. A related model is suggested by the fact that CER2-LIKEs are homologous to BAHD acyltransferases, which bind acyl-CoA substrates; if CER2-LIKEs have retained their ancestral capacity to bind acyl-CoAs, they may physically extend the substrate-binding pocket of the condensing enzyme. CER2-LIKEs could thereby determine the maximum length of acyl-CoA accepted for condensation with malonyl-CoA.

Acyl-CoAs are amphipathic molecules that partition into lipid membranes [42,43], with membrane affinity increasing with chain length [44]. It is not known how substrate is received and transferred between components of the fatty acid elongase, within or between elongation cycles. In Arabidopsis, cuticular lipids constitute roughly 50% of the acyl-lipid output of stem epidermal cells [45], and the 28-, 30- and 32-carbon acyl-CoA precursors of cuticular waxes make up nearly 20% of the acyl-CoA pool of whole rosette leaves [24]. Such a large pool of long, saturated lipids could potentially disrupt ER membrane integrity. Association of VLC-acyl-CoA precursors of cuticular waxes with acyl-CoA binding proteins could reduce acyl-CoA partitioning into the membrane, and thus, membrane damage. The Arabidopsis acyl-CoA binding protein (ACBP) family is known to bind acyl-CoAs in the cytosol, making them available for transport and lipid synthesis, and preventing hydrolysis of the CoA group [46]. However, no members of this protein family have been shown to work with acyl chain lengths in excess of 26 carbons [47]. If CER2-LIKE proteins do bind acyl-CoAs, they might serve as specialized VLC-acyl-CoA binding proteins. Since CER2 interacts with CER6, CER2-LIKEs could also serve to concentrate substrate around the fatty acid elongase complex, and specificities of CER2-LIKEs for different chain lengths of acyl-CoAs could determine what substrates would be available for use by the condensing enzyme. Unlike the previous two models, this one implies that CER6 constitutively accepts substrate chain-lengths of acyl-CoAs up to 32 carbons in length, but is limited by the availability of these VLC-acyl-CoAs.

Recent work on the Atf1p alcohol acetyltransferase of *Saccharomyces cerevisiae* has suggested a final model for CER2-LIKE function. Atf1p has low sequence identity to plant BAHD acyltransferases, and shares the HXXXD motif described above [48,49]. Intriguingly, in addition to acyl transfer activity, Atf1p has thioesterase activity *in vitro*. Though thioester bond cleavage is intrinsic to the acyl transfer reaction shown in Figure 3, Atf1p was shown to have thioesterase activity in the absence of an acyl donor, indicating that this is a distinct catalytic activity. Further, mutagenesis of the predicted catalytic histidine residue of the HXXXD motif reduced, but did not abolish, thioesterase activity of Atf1p [49]. It is possible that related proteins, including CER2-LIKEs, could also have thioesterase activity. As thioester cleavage is a necessary step within the process of condensation, it is possible that CER2-LIKEs release acyl groups from CoA to assist in substrate loading onto the active-site cysteine residue of the condensing enzyme.

## 5. The *CER2-LIKE* Gene Family

BAHDs are found in plants and fungi, and function in diverse, specialized metabolic pathways to synthesize a wide variety of products. The BAHD family was named after its first four characterized members: benzylalcohol *O*-acetyltransferase from *Clarkia breweri* (BEAT); anthocyanin *O*-hydroxycinnamoyltransferases (AHCTs) from *Petunia*, *Senecio*, *Gentiana*, *Perilla*, and *Lavandula*; anthranilate *N*-hydroxycinnamoyl/benzoyltransferase from *Dianthus caryophyllus* (HCBT); and deacetylvindoline 4-*O*-acetyltransferase (DAT) from *Catharanthus roseus* [48]. BAHD acyltransferases are pervasive throughout the plant kingdom [50]. The distribution of *CER2-LIKE*s, however, is more difficult to ascertain. *CER2-LIKE*s are homologous to, but functionally distinct from, BAHD acyltransferases. To date, five *CER2-LIKE* genes from Arabidopsis, maize *GLOSSY2*, and a *CER2-LIKE* from *Oryza sativa* [28] have been identified, characterized, and shown to have analogous effects on the elongation of VLCFA precursors of waxes. Phylogenetic studies have also identified *CER2* homologs in *Populus* [51,52], *Vitis*, and *Medicago* [52] based on their clustering with Arabidopsis *CER2* in wider analyses of the BAHD acyltransferase superfamily. It was recently reported that there are no *CER2* homologs in the bryophyte *Funaria hygrometrica* [53], nor in the charyophycean alga *Klebsormidium flaccidum* [54]. Together, these findings suggest that CER2-LIKE function may have been acquired in the vascular plant lineage.

**Figure 5:**
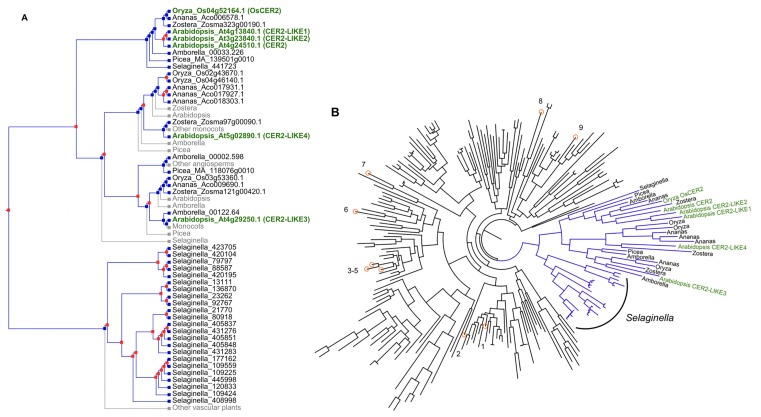
(**A**) Phylogenetic reconstruction of inferred gene duplications (red boxes) and losses (grey) in the smallest clade of BAHD acyltransferases that includes all five Arabidopsis CER2-LIKE homologs and their closest relatives (a large clade of *Selaginella* homologs). Some inferred losses affect the entire clades noted. Biochemically characterized CER2-LIKEs are in green text; (**B**) Phylogram of the BAHD acyltransferases recovered using Arabidopsis CER2-LIKE homologs. The subclade in blue was used for the tree-reconciliation analysis (**A**). Characterized BAHD acyltransferases from Arabidopsis are numbered, clockwise, and marked on the tree with orange circles; (1) At5g48930, HCT/HYDROXYCINNAMOYL-COA SHIKIMATE/QUINATE HYDROXYCINNAMOYL TRANSFERASE [62]; (2) At2g19070, SHT/SPERMIDINE HYDROXYCINNAMOYLTRANSFERASE [63]; (3) At5g41040, ASFT/ALIPHATIC SUBERIN FERULOYL TRANSFERASE [64]; (4) At3g48720, DCF/DEFICIENT IN CUTIN FERULATE [32]; (5) At5g63560, FACT/FATTY ALCOHOL:CAFFEOYL-COA CAFFEOYL TRANSFERASE [65]; (6) At1g65450, GLC/GLAUCE [66]; (7) At4g31910, DRL/DWARF AND ROUND LEAF 1 [67]/BAT1/BR-RELATED-ACYLTRANSFERASE [68]/PIZ/PIZZA [69]; (8) At3g29670, PMAT2/PHENOLIC GLUCOSIDE MALONYLTRANSFERASE 2 [70]; (9) At5g67160, EPS2/ENHANCED PSEUDOMONAS SUSCEPTIBILITY 1 [71]. The full gene tree (with complete gene identifiers and scale) is provided in the supplemental information.

To better assess the distribution of *CER2-LIKE* genes across the plant kingdom, we examined the phylogeny of the entire BAHD acyltransferase family in diverse plant species (Figure 5). *Klebsormidium flaccidum* was included in our analysis as a representative charophycean alga. *Klebsormidium* is often found in semi-aquatic environments and has an extracellular lipidic capsule that protects it from desiccation. The capsule is, however, composed of different lipids than those found in plant cuticles [54]. The liverwort *Marchantia polymorpha* and mosses *Physcomitrella patens* and *Sphagnum fallax* were selected for analysis as representative bryophytes. The lycophyte *Selaginella moellendorffii* was included to represent early-diverged vascular plants, and *Picea abies* as a representative gymnosperm. *Amborella trichopoda*, *Arabidopsis thaliana*, *Oryza sativa*, *Ananas comosus*, and *Zostera marina* were selected to provide a broad sampling of angiosperms. *Amborella* was of particular interest due to its position as the sister group to all other angiosperms, and *Zostera* was of interest as an aquatic plant species. The full method, the genomic resources used, sequence alignment, and complete phylogram are published as supplemental material.

Genes with homology to BAHD acyltransferases were identified in the genomes of all the organisms we selected for analysis, including the green alga *Klebsormidium* (Supplemental Figure S1). The smallest clade that contained all of the characterized Arabidopsis *CER2-LIKE* homologs also included sequences from all of the selected vascular plant species, but no bryophyte or green algal sequences (Figure 5A). We designate this group of sequences as a putative “*CER2-LIKE* clade”, nested within the BAHD acyltransferase superfamily and corresponding to the previously defined BAHD acyltransferase clade II [30]. Our designation is based entirely on phylogeny, and functional analyses will be required to determine whether the candidate genes identified here encode true CER2-LIKEs, BAHD acyltransferases, or neither. Candidate *CER2-LIKE*s could be characterized by heterologous expression in yeast, complementation of Arabidopsis mutants, and, where possible, by mutant analysis. A sequence identical to OsCER*2* was also identified in our homology-based search; however, this gene is designated Os04g52164.1 according to *Oryza sativa v7_JGI*, while OsCER*2* was previously reported to be Os04g0611200 [28].

The BAHD superfamily as a whole has a high degree of sequence divergence. Calculation of the sequence identity among characterized Arabidopsis CER2-LIKEs revealed that this can be observed even within clades with related biochemical function. For example, Arabidopsis CER2 has approximately 35% identity with CER2-LIKE1 and CER2-LIKE2, and only 20% identity with CER2-LIKE3 and CER2-LIKE4. By comparison, CER2 has approximately 18% identity to three randomly-selected, characterized Arabidopsis BAHDs outside of the “CER2-LIKE” clade; the selected BAHDs were EPS1, PMAT2, and ASFT (indicated in Figure 5B).

All of the putative *CER2-LIKEs* that we identified are the products of gene duplications that occurred exclusively within vascular plants. Additional homologs from many of these vascular plant lineages are implied to have existed, but are inferred to have been lost (Figure 5A). A major set of gene duplications occurred separately in *Selaginella*, leading to a very large clade of *Selaginella* genes (Figure 5A,B) sister to the *CER2-LIKE* clade. Whether proteins encoded by these genes participate in fatty acid elongation of cuticular wax precursors, analogous to angiosperm CER2-LIKEs, will be particularly interesting to investigate. Wax composition and cuticle structure have not been studied in *Selaginella* nor in any lycophyte as far as we are aware. The closest related species with known wax composition are the ferns *Osmunda regalis* [55] and two *Pteridium* (bracken fern) species [56].

That the *CER2-LIKE* clade did not include sequences from *Klebsormidium*, *Marchantia*, *Physcomitrella*, or *Sphagnum* supports the assertion that CER2-LIKE function could be a derived trait of vascular plants. Our sample size is currently small, and this idea will be tested as genomic resources for more diverse green algae and bryophytes become available. Notably, the described function of CER2-LIKEs may not be limited to the clade defined here; further investigation may reveal proteins homologous or non-homologous to CER2-LIKEs that fulfill similar metabolic roles. However, the proposition that *CER2-LIKE*s could be absent from bryophytes is consistent with the observation that many bryophyte gametophytes synthesize cuticular waxes of shorter average chain lengths compared to vascular plants. In the gametophyte cuticle of the model moss *Physcomitrella patens* (*P. patens*), the most abundant waxes are primary alcohols and VLCFAs 24, 26, and 28 carbons in length, and wax esters 42–50 carbons in length (dimers of a fatty acid and a fatty alcohol) [57]. Similar chain length composition is observed across gametophytes of twelve species of the peat bog moss genus *Sphagnum* [58]. A study that examined cuticular waxes of both endohydric (*Pogonatum*) and ectohydric (*Andreaea*) gametophytes had similar results, with 22–28 carbon waxes predominating [59]. Analysis of *Pogonatum* sporophyte cuticular wax, however, revealed 29-carbon alkanols as the most abundant wax component [60]. Waxes longer than 28 carbons are also found in the cuticles of moss gametophytes; they are simply less abundant than the shorter components in many characterized species. Therefore, if it is true that CER2-LIKEs are a derived feature of vascular plants, bryophytes must have alternative mechanisms for synthesizing 30-carbon VLCFAs, for example, specialized KCS or ELO condensing enzymes.

The importance of CER2-LIKEs for cuticular wax synthesis is obvious in species such as Arabidopsis and maize where *cer2-like* mutants have been characterized. It is evident from the above discussion of bryophytes, however, that it is far more difficult to establish the contribution of this gene family to plant cuticular wax metabolism in a general sense. Moreover, the significance of wax chain length for cuticle properties is ambiguous. Like bryophytes, many grasses also accumulate relatively short waxes (excluding wax esters), but exhibit conspicuous wax blooms that function as robust water barriers [61]. This clearly indicates that wax chain length does not define cuticle functionality. At present, it is only possible to conclude that waxes derived from VLCFAs with the greatest chain lengths are important components of some plant cuticles, and that CER2-LIKEs are required for efficient production of these VLCFA wax precursors in some vascular plants.

## 6. Conclusions

The elongation of very-long-chain fatty acid precursors is a necessary and dedicated step in cuticular wax biosynthesis. In addition to the core elongation machinery, CER2-LIKE proteins contribute to this process and determine the chain length profile of elongation products. The precise biochemical function of CER2-LIKEs remains unknown, but multiple lines of evidence indicate that it is distinct from the role described for the BAHD acyltransferase family of proteins from which CER2-LIKEs are derived. Five *CER2-LIKE* genes have been studied from Arabidopsis and one gene each from rice and maize. Molecular genetic characterization of homologous genes/proteins from more diverse plant lineages will be necessary to understand both the importance of the CER2-LIKE protein family for the evolution of cuticular barriers, and the surprising specialization of the CER2-LIKE clade within the BAHD acyltransferase family.

## Supplementary Materials

Supplementary materials are available online at www.mdpi.com/2223-7747/6/2/23/s1.

## References

[1] Fich E.A., Segerson N.A., Rose J.K.C. (2016). The plant polyester cutin: Biosynthesis, structure, and biological roles. Annu. Rev. Plant Biol..

[2] Yeats T.H., Rose J.K.C. (2013). The formation and function of plant cuticles. Plant Physiol..

[3] Haslam T.M., Kunst L. (2013). Extending the story of very-long-chain fatty acid elongation. Plant Sci..

[4] Millar A.A., Kunst L. (1997). Very-long-chain fatty acid biosynthesis is controlled through the expression and specificity of the condensing enzyme. Plant J..

[5] Bach L., Michaelson L.V., Haslam R., Bellec Y., Gissot L., Marion J., Da Costa M., Boutin J.P., Miquel M., Tellier F. (2008). The very-long-chain hydroxy fatty acyl-CoA dehydratase PASTICCINO2 is essential and limiting for plant development. Proc. Natl. Acad. Sci. USA.

[6] Morineau C., Gissot L., Bellec Y., Hematy K., Tellier F., Renne C., Haslam R., Beaudoin F., Napier J., Faure J.D. (2016). Dual fatty acid elongase complex interactions in Arabidopsis. PLoS ONE.

[7] Beaudoin F., Wu X., Li F., Haslam R.P., Markham J.E., Zheng H., Napier J.A., Kunst L. (2009). Functional characterization of the Arabidopsis β-ketoacyl-coenzyme A reductase candidates of the fatty acid elongase. Plant Physiol..

[8] Joubès J., Raffaele S., Bourdenx B., Garcia C., Laroche-Traineau J., Moreau P., Domergue F., Lessire R. (2008). The VLCFA elongase gene family in *Arabidopsis thaliana*: Phylogenetic analysis, 3D modelling and expression profiling. Plant Mol. Biol..

[9] Zank T.K., Zähringer U., Beckmann C., Pohnert G., Boland W., Holtorf H., Reski R., Lerchl J., Heinz E. (2002). Cloning and functional characterisation of an enzyme involved in the elongation of ∆6-polyunsaturated fatty acids from the moss *Physcomitrella patens*. Plant J..

[10] Eiamsa-Ard P., Kanjana-Opas A., Cahoon E.B., Chodok P., Kaewsuwan S. (2013). Two novel *Physcomitrella patens* fatty acid elongases (ELOs): Identification and functional characterization. Appl. Microbiol. Biotech..

[11] Trenkamp S., Martin W., Tietjen K. (2004). Specific and differential inhibition of very-long-chain fatty acid elongases from *Arabidopsis thaliana* by different herbicides. Proc. Natl. Acad. Sci. USA.

[12] Blacklock B.J., Jaworski J.G. (2006). Substrate specificity of Arabidopsis 3-ketoacyl-CoA synthases. Biochem. Biophys. Res. Commun..

[13] Paul S., Gable K., Beaudoin F., Cahoon E., Jaworski J., Napier J.A., Dunn T.M. (2006). Members of the Arabidopsis *FAE1-like* 3-ketoacyl-CoA synthase gene family substitute for the Elop proteins of *Saccharomyces cerevisiae*. J. Biol. Chem..

[14] Tresch S., Heilmann M., Christiansen N., Looser R., Grossmann K. (2012). Inhibition of saturated very-long-chain fatty acid biosynthesis by mefluidide and perfluidone, selective inhibitors of 3-ketoacyl-CoA synthases. Phytochemistry.

[15] Koornneef M., Hanhart C.J., Thiel F. (1989). A genetic and phenotypic description of *eceriferum* (*cer*) mutants in *Arabidopsis thaliana*. J. Hered..

[16] McNevin J.P., Woodward W., Hannoufa A., Feldmann K.A., Lemieux B. (1993). Isolation and characterization of *eceriferum* (*cer*) mutants induced by T-DNA insertions in *Arabidopsis thaliana*. Genome.

[17] Negruk V., Yang P., Subramanian M., McNevin J.P., Lemieux B. (1996). Molecular cloning and characterization of the *CER*2 gene of *Arabidopsis thaliana*. Plant J..

[18] Xia Y., Nikolau B.J., Schnable P.S. (1996). Cloning and characterization of *CER*2, an Arabidopsis gene that affects cuticular wax accumulation. Plant Cell.

[19] Millar A.A., Clemens S., Zachgo S., Giblin E.M., Taylor D.C., Kunst L. (1999). *CUT1*, an Arabidopsis gene required for cuticular wax biosynthesis and pollen fertility, encodes a very-long-chain fatty acid condensing enzyme. Plant Cell.

[20] Haslam T.M., Manas-Fernandez A., Zhao L., Kunst L. (2012). Arabidopsis ECERIFERUM2 is a component of the fatty acid elongation machinery required for fatty acid extension to exceptional lengths. Plant Physiol..

[21] Bianchi G., Avato P., Salamini F. (1975). *Glossy* mutants of maize. VI. Chemical constituents of *glossy-2* epicuticular waxes. Maydica.

[22] Tacke E., Korfhage C., Michel D., Maddaloni M., Motto M., Lanzini S., Salamini F., Doring H.P. (1995). Transposon tagging of the maize *Glossy2* locus with the transposable element *En/Spm*. Plant J..

[23] Pascal S., Bernard A., Sorel M., Pervent M., Vile D., Haslam R.P., Napier J.A., Lessire R., Domergue F., Joubès J. (2013). The Arabidopsis *cer*26 mutant, like the *cer*2 mutant, is specifically affected in the very long chain fatty acid elongation process. Plant J..

[24] Haslam T.M., Haslam R., Thoraval D., Pascal S., Delude C., Domergue F., Fernández A.M., Beaudoin F., Napier J.A., Kunst L. (2015). ECERIFERUM2-LIKE proteins have unique biochemical and physiological functions in very-long-chain fatty acid elongation. Plant Physiol..

[25] Haslam T.M., Kunst L. (2017). Arabidopsis ECERIFERUM2-LIKEs are mediators of condensing enzyme function. Plant Physiol.

[26] Preuss D., Lemieux B., Yen G., Davis R.W. (1993). A conditional sterile mutation eliminates surface components from Arabidopsis pollen and disrupts cell signaling during fertilization. Genes Dev..

[27] Hegebarth D., Buschhaus C., Joubès J., Thoraval D., Bird D., Jetter R. (2007). Arabidopsis ketoacyl-CoA synthase 16 forms C36/C38 acyl precursors for leaf trichome and pavement surface wax. Plant. Cell Environ..

[28] Wang X., Guan Y., Zhang D., Dong X., Tian L., Qu L.Q. (2017). A β-ketoacyl-CoA synthase is involved in rice leaf cuticular wax synthesis and requires a CER2-LIKE protein as a cofactor. Plant Physiol..

[29] Xu L., Zeisler V., Schreiber L., Gao J., Hu K., Wen J., Yi B. (2017). Overexpression of the novel Arabidopsis gene At5g02890 alters inflorescence stem wax composition and affects phytohormone homeostasis. Front. Plant Sci..

[30] D’Auria J.C. (2006). Acyltransferases in plants: A good time to be BAHD. Curr. Opin. Plant Biol..

[31] Panikashvili D., Shi J.X., Schreiber L., Aharoni A. (2009). The Arabidopsis *DCR* encoding a soluble BAHD acyltransferase is required for cutin polyester formation and seed hydration properties. Plant Physiol..

[32] Rautengarten C., Ebert B., Ouellet M., Nafisi M., Baidoo E.E.K., Benke P., Stranne M., Mukhopadhyay A., Keasling J.D., Sakuragi Y. (2012). Arabidopsis *deficient in cutin ferulate* encodes a transferase required for feruloylation of ω-hydroxy fatty acids in cutin polyester. Plant Physiol..

[33] Suzuki H., Nakayama T., Nishino T. (2003). Proposed mechanism and functional amino acid residues of malonyl-CoA: anthocyanin 5-*O*-glucoside-6’’’-*O*-malonyltransferase from flowers of *Salvia splendens*, a member of the versatile plant acyltransferase family. Biochemistry.

[34] Bayer A., Ma X., Stöckigt J. (2004). Acetyltransfer in natural product biosynthesis—Functional cloning and molecular analysis of vinorine synthase. Bioorg. Med. Chem..

[35] Lallemand L.A., Zubieta C., Lee S.G., Wang Y., Acajjaoui S., Timmins J., McSweeney S., Jez J.M., McCarthy J.G., McCarthy A.A. (2012). A structural basis for the biosynthesis of the major chlorogenic acids found in coffee. Plant Physiol..

[36] Walker A.M., Hayes R.P., Youn B., Vermerris W., Sattler S.E., Kang C. (2013). Elucidation of the structure and reaction mechanism of sorghum hydroxycinnamoyltransferase and its structural relationship to other coenzyme A-dependent transferases and synthases. Plant Physiol..

[37] Ma X., Koepke J., Panjikar S., Fritzsch G., Stöckigt J. (2005). Crystal structure of vinorine synthase, the first representative of the BAHD superfamily. J. Biol. Chem..

[38] Unno H., Ichimaida F., Suzuki H., Takahashi S., Tanaka Y., Saito A., Nishino T., Kusunoki M., Nakayama T. (2007). Structural and mutational studies of anthocyanin malonyltransferases establish the features of BAHD enzyme catalysis. J. Biol. Chem..

[39] Morales-Quintana L., Nuñez-Tobar M.X., Moya-León M.A., Herrera R. (2013). Molecular dynamics simulation and site-directed mutagenesis of alcohol acyltransferase: A proposed mechanism of catalysis. J. Chem. Inf. Model..

[40] Blacklock B.J., Jaworski J.G. (2002). Studies into factors contributing to substrate specificity of membrane-bound 3-ketoacyl-CoA synthases. Eur. J. Biochem..

[41] Jasinski S., Lecureuil A., Miquel M., Loudet O., Raffaele S., Froissard M., Guerche P. (2012). Natural variation in seed very long chain fatty acid content is controlled by a new isoform of KCS18 in *Arabidopsis thaliana*. PLoS ONE.

[42] Jolly C.A., Hubbell T., Behnke W.D., Schroeder F. (1997). Fatty acid binding protein: Stimulation of microsomal phosphatidic acid formation. Arch. Biochem. Biophys..

[43] Gossett R.E., Frolov A.A., Roths J.B., Behnke W.D., Kier A.B., Schroeder F. (1996). Acyl-CoA binding proteins: Multiplicity and function. Lipids.

[44] Boylan J.G., Hamilton J.A. (1992). Interactions of acyl-coenzyme A with phosphatidylcholine bilayers and serum albumin. Biochemistry.

[45] Suh M.C., Samuels A.L., Jetter R., Kunst L., Pollard M., Ohlrogge J., Beisson F. (2005). Cuticular lipid composition, surface structure, and gene expression in Arabidopsis stem epidermis. Plant Physiol..

[46] Xiao S., Chye M.L. (2011). New roles for acyl-CoA-binding proteins (ACBPs) in plant development, stress responses and lipid metabolism. Prog. Lipid Res..

[47] Xue Y., Xiao S., Kim J., Lung S., Chen L., Tanner J.A., Suh M.C., Chye M.L. (2014). Arabidopsis membrane-associated acyl-CoA-binding protein ACBP1 is involved in stem cuticle formation. J. Exp. Bot..

[48] St-Pierre B., Luca V.D. (2000). Evolution of acyltransferase genes: Origin and diversification of the BAHD superfamily of acyltransferase involved in secondary metabolism. Evol. Metab. Pathw..

[49] Nancolas B., Bull I., Stenner R., Dufour V., Curnow P. (2017). *Saccharomyces cerevisiae* Atf1p is an alcohol acetyltransferase and a thioesterase *in vitro*. Yeast.

[50] Bartley L.E., Peck M.L., Kim S.R., Ebert B., Maniseri C., Chiniquy D., Sykes R., Gao L., Rautengarten C., Vega-Sanchez M.E. (2013). Overexpression of a BAHD acyltransferase, OsAt10, alters rice cell wall hydroxycinnamic acid content and saccharification. Plant Physiol..

[51] Yu X.H., Gou J.Y., Liu C.J. (2009). BAHD superfamily of acyl-CoA dependent acyltransferases in *Populus* and Arabidopsis: Bioinformatics and gene expression. Plant Mol. Biol..

[52] Tuominen L.K., Johnson V.E., Tsai C.J. (2011). Differential phylogenetic expansions in BAHD acyltransferases across five angiosperm taxa and evidence of divergent expression among *Populus* paralogues. BMC Genom..

[53] Busta L., Budke J.M., Jetter R. (2016). The moss *Funaria hygrometrica* has cuticular wax similar to vascular plants, with distinct composition on leafy gametophyte, calyptra and sporophyte capsule surfaces. Ann. Bot..

[54] Kondo S., Hori K., Sasaki-Sekimoto Y., Kobayashi A., Kato T., Yuno-Ohta N., Nobusawa T., Ohtaka K., Shimojima M., Ohta H. (2016). Primitive extracellular lipid components on the surface of the charophytic alga *Klebsormidium flaccidum* and their possible biosynthetic pathways as deduced from the genome sequence. Front. Plant Sci..

[55] Jetter R., Riederer M. (2000). Composition of cuticular waxes on *Osmunda regalis* fronds. J. Chem. Ecol..

[56] Baker E.A., Gaskin R.E. (1987). Composition of leaf epicuticular waxes of *Pteridium* sub-species. Phytochemistry.

[57] Buda G.J., Barnes W.J., Fich E.A., Park S., Yeats T.H., Zhao L., Domozych D.S., Rose J.K.C. (2013). An ATP binding cassette transporter is required for cuticular wax deposition and desiccation tolerance in the moss *Physcomitrella patens*. Plant Cell.

[58] Baas M., Pancost R., van Geel B., Sinninghe Damsté J.S. (2000). A comparative study of lipids in *Sphagnum* species. Org. Geochem..

[59] Haas K. (1982). Surface wax of *Andreaea* and *Pogonatum* species. Phytochemistry.

[60] Neinhuis C., Jetter R. (1995). Ultrastructure and chemistry of epicuticular wax crystals in Polytrichales sporophytes. J. Bryol..

[61] Richardson A., Wojciechowski T., Franke R., Schreiber L., Kerstiens G., Jarvis M., Fricke W. (2007). Cuticular permeance in relation to wax and cutin development along the growing barley (*Hordeum vulgare*) leaf. Planta.

[62] Hoffmann L., Bessau S., Geoffroy P., Ritzenthaler C., Meyer D., Lapierre C., Pollet B., Legrand M. (2004). Silencing of hydroxycinnamoyl-coenzyme A shikimate/quinate hydroxycinnamoyltransferase affects phenylpropanoid biosynthesis. Plant Cell.

[63] Grienenberger E., Besseau S., Geoffroy P., Debayle D., Heintz D., Lapierre C., Pollet B., Heitz T., Legrand M. (2009). A BAHD acyltransferase is expressed in the tapetum of Arabidopsis anthers and is involved in the synthesis of hydroxycinnamoyl spermidines. Plant J..

[64] Molina I., Li-Beisson Y., Beisson F., Ohlrogge J.B., Pollard M. (2009). Identification of an Arabidopsis feruloyl-coenzyme A transferase required for suberin synthesis. Plant Physiol..

[65] Kosma D.K., Molina I., Ohlrogge J.B., Pollard M. (2012). Identification of an Arabidopsis fatty alcohol: caffeoyl-coenzyme A acyltransferase required for the synthesis of alkyl hydroxycinnamates in root waxes. Plant Physiol..

[66] Leshem Y., Johnson C., Wuest S.E., Song X., Ngo Q.A., Grossniklaus U., Sundaresan V. (2012). Molecular characterization of the *glauce* mutant: A central cell-specific function is required for double fertilization in Arabidopsis. Plant Cell.

[67] Zhu W., Wang H., Fujioka S., Zhou T., Tian H., Tian W., Wang X. (2013). Homeostasis of brassinosteroids regulated by DRL1, a putative acyltransferase in Arabidopsis. Mol. Plant.

[68] Choi S., Cho Y.H., Kim K., Matsui M., Son S.H., Kim S.K., Fujioka S., Hwang I. (2013). BAT1, a putative acyltransferase, modulates brassinosteroid levels in Arabidopsis. Plant J..

[69] Schneider K., Breuer C., Kawamura A., Jikumaru Y., Hanada A., Fujioka S., Ichikawa T., Kondou Y., Matsui M., Kamiya Y. (2012). Arabidopsis PIZZA has the capacity to acylate brassinosteroids. PLoS ONE.

[70] Taguchi G., Ubukata T., Nozue H., Kobayashi Y., Takahi M., Yamamoto H., Hayashida N. (2010). Malonylation is a key reaction in the metabolism of xenobiotic phenolic glucosides in Arabidopsis and tobacco. Plant J..

[71] Zheng Z., Qualley A., Fan B., Dudareva N., Chen Z. (2009). An important role of a BAHD acyl transferase-like protein in plant innate immunity. Plant J..

