# Supplementary material for: The Unique Role of the ECERIFERUM2-LIKE Clade of the BAHD Acyltransferase Superfamily in Cuticular Wax Metabolism
Source: Plants (Basel). 2017 Jun 13;6(2):23. doi: 10.3390/plants6020023 (PMC5489795; doi:10.3390/plants6020023)
Supplement: Supplementary file 1 [file plants-06-00023-s001.zip › Haslam&al2017_Supplementary Methods_27Apr17.pdf]

Supplementary Methods

We retrieved BAHD acyltransferases using the five *CER2*-LIKE homologs from Arabidopsis with similarity searches using available genomic resources. As we were primarily interested in addressing *CER2*-related gene duplications that may predate angiosperm evolution, we focused our taxon sampling on non-flowering land-plant genomes available on Phytozome v. 12 [1] (i.e., *Marchantia polymorpha v3.1*; *Physcomitrella patens v3.3*; *Sphagnum fallax v0.5*; *Selaginella moellendorffii v1.0*) and several representative angiosperms (*Amborella trichopoda v1.0*; *Oryza sativa v7_JGI*; *Ananas comosus v3*; *Zostera marina v2.2*; *Arabidopsis thaliana TAIR10*); we also surveyed a conifer genome (*Picea abies v1.0 complete*) and a charophyte green algal genome (*Klebsormidium flaccidum V1.0*) from congenie.org and plantmorphogenesis.bio.titech.ac.jp, respectively. For sequences on Phytozome we used the "protein homologs" tool (under gene view), which considers the query sequence against all other proteins on Phytozome, retaining all hits; we repeated this search for each of the Arabidopsis homologs in turn. For *Klebsormidium* and *Picea*, we searched the respective database for each homolog using tblastx with default settings, retaining all hits. We aligned all recovered amino-acid sequences using MUSCLE v3.8.31 [2], and performed maximum-likelihood phylogenetic inference using RAxML v8.2.4 [3] with the VT+G amino acid substitution model (model selected using PartitionFinderProtein v2.1.1 [4] with the Bayesian Information Criterion), considering 20 starting trees. We estimated branch support using 500 rapid bootstrap replicates (bootstrap values mapped onto the best tree using SumTrees v4.0.0 [5]). We then reconciled a large gene-tree clade in the resulting analysis (the least-inclusive clade containing all Arabidopsis CER2 homologs, in addition to their sister-group, a large clade of *Selaginella* homologs) with a well-accepted species tree for vascular plants [(*Selaginella*, (*Picea*, (*Amborella*, (*Arabidopsis*, (*Zostera*, (*Ananas*, *Oryza*))))))], using Notung v2.9 [6] with default settings.

Supplementary Literature Cited

1. Goodstein, D. M.; Shu, S.; Howson, R.; Neupane, R.; Hayes, R. D.; Fazo, J.; Mitros, T.; Dirks, W.; Hellsten, U.; Putnam, N.; Rokhsar, D. S. Phytozome : a comparative platform for green plant genomics. 2012, 40, 1178–1186.

2. Edgar, R. C.; Drive, R. M.; Valley, M. MUSCLE : multiple sequence alignment with high accuracy and high throughput. 2004, 32, 1792–1797.

3. Stamatakis, A. RAxML version 8 : a tool for phylogenetic analysis and post-analysis of large phylogenies. 2014, 30, 1312–1313.

4. Lanfear, R.; Frandsen, P. B.; Wright, A. M.; Senfeld, T.; Calcott, B. PartitionFinder 2 : New Methods for Selecting Partitioned Models of Evolution for Molecular and Morphological Phylogenetic Analyses. 2016, 34, 772–773.

5. Sukumaran, J.; Holder, M. T. DendroPy : a Python library for phylogenetic computing. 2010, 26, 1569–1571.

6. Stolzer, M.; Lai, H.; Xu, M.; Sathaye, D.; Vernot, B.; Durand, D. Inferring duplications , losses , transfers and incomplete lineage sorting with nonbinary species trees. 2012, 28, 409–415.
