# Supplementary figures and images for: The Unique Role of the ECERIFERUM2-LIKE Clade of the BAHD Acyltransferase Superfamily in Cuticular Wax Metabolism
Source: Plants (Basel). 2017 Jun 13;6(2):23. doi: 10.3390/plants6020023 (PMC5489795; doi:10.3390/plants6020023)

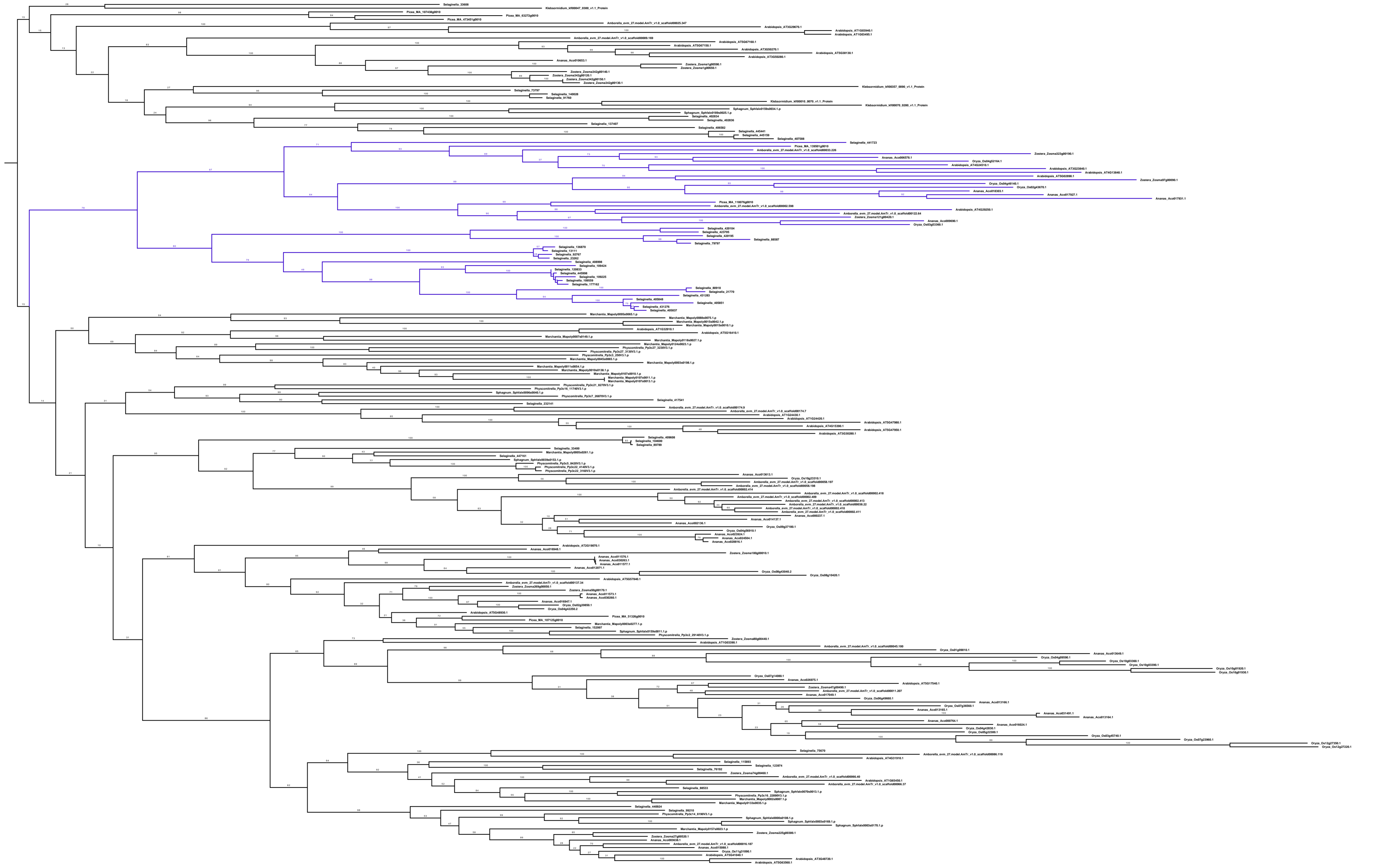

Supplement: Supplementary file 1 [file plants-06-00023-s001.zip › Haslam&al_Supplementary_BAHD_acylttransferase_full_tree_27Apr17.pdf]
